# *In situ* hybridization protocol for enhanced detection of gene expression in the planarian *Schmidtea mediterranea*

**DOI:** 10.1186/1471-213X-13-8

**Published:** 2013-03-12

**Authors:** Ryan S King, Phillip A Newmark

**Affiliations:** 1 Howard Hughes Medical Institute, Department of Cell and Developmental Biology, University of Illinois at Urbana-Champaign, 601 South Goodwin Avenue, Urbana, IL 61801, USA

**Keywords:** Planarian, Whole-mount *in situ* hybridization (WISH), Fluorescent *in situ* hybridization (FISH), Tyramide signal amplification (TSA), Autofluorescence, Multicolor FISH, Peroxidase quenching, Regeneration, Heat-induced antigen retrieval (HIAR)

## Abstract

**Background:**

The freshwater planarian *Schmidtea mediterranea* has emerged as a powerful model for studies of regenerative, stem cell, and germ cell biology. Whole-mount *in situ* hybridization (WISH) and whole-mount fluorescent *in situ* hybridization (FISH) are critical methods for determining gene expression patterns in planarians. While expression patterns for a number of genes have been elucidated using established protocols, determining the expression patterns for particularly low-abundance transcripts remains a challenge.

**Results:**

We show here that a short bleaching step in formamide dramatically enhances signal intensity of WISH and FISH. To further improve signal sensitivity we optimized blocking conditions for multiple anti-hapten antibodies, developed a copper sulfate quenching step that virtually eliminates autofluorescence, and enhanced signal intensity through iterative rounds of tyramide signal amplification. For FISH on regenerating planarians, we employed a heat-induced antigen retrieval step that provides a better balance between permeabilization of mature tissues and preservation of regenerating tissues. We also show that azide most effectively quenches peroxidase activity between rounds of development for multicolor FISH experiments. Finally, we apply these modifications to elucidate the expression patterns of a few low-abundance transcripts.

**Conclusion:**

The modifications we present here provide significant improvements in signal intensity and signal sensitivity for WISH and FISH in planarians. Additionally, these modifications might be of widespread utility for whole-mount FISH in other model organisms.

## Background

Planarians are re-emerging as a choice animal model for studying regeneration, with the recent development of genomic resources and molecular tools in a few species, including *Schmidtea mediterranea* and *Dugesia japonica*[1]. After captivating scientists with their remarkable regenerative capacity for over a century [2], significant progress is being made in understanding how planarians reestablish axial polarity following injury [3-5], how their stem cells regulate choices between self-renewal and differentiation [6,7], and how their organ systems, including the central nervous system [8-11], intestine [12,13], excretory [14,15], and reproductive system [16-19], regenerate following injury.

Whole-mount *in situ* hybridization (WISH) and whole-mount fluorescent *in situ* hybridization (FISH) are critical techniques for determining gene expression patterns. Planarians present several challenges for (F)ISH: first, planarians secrete a layer of mucous that needs to be removed prior to fixation; second, some planarian tissues are “sticky”, resulting in non-specific binding or trapping of antibodies used for detection; third, planarian tissue autofluoresces across a broad range of wavelengths leading to a poor signal-to-noise ratio for low-abundance genes by FISH; and fourth, regenerating tissue is fragile during early stages of regeneration, necessitating a fine balance during permeabilization to allow even probe penetration of mature tissues while preserving morphology of regenerating tissue.

Early WISH protocols in planarians utilized treatment with hydrochloric acid and alcohol-based fixation to overcome issues with planarian mucous [20]. More recently a formaldehyde-based WISH protocol, which utilizes N-acetyl-cysteine for mucous removal, was developed, providing vastly better sensitivity and maintenance of morphology for WISH of planarians [21]. This protocol has been widely utilized and has been a critical advancement in the field. However, as with other model organisms, elucidation of the expression patterns for low-abundance transcripts remains challenging. In some cases the expression patterns for genes with known functions remain unclear or elusive [15,22,23].

Fluorescent detection of transcripts provides superior spatial resolution and enables visualization of overlapping expression patterns compared to development with chromogenic substrates. While the recently developed formaldehyde-based WISH protocol does provide improved signal sensitivity for FISH, we further improved signal sensitivity by systematically optimizing several key steps, including the bleaching, blocking, and washing steps. Furthermore, multicolor FISH using tyramide signal amplification (TSA) requires sequential rounds of amplification using peroxidase-conjugated reagents. Therefore, to prevent residual peroxidase activity from generating false signal during subsequent rounds of detection it is critical to efficiently quench peroxidase activity between TSA reactions. We directly compared several methods for quenching peroxidase activity and find that incubation with azide is the most effective at quenching peroxidase activity and the least detrimental toward detection of gene expression in subsequent rounds of TSA. These modifications represent a significant improvement for FISH in planarians, and we have utilized these advancements to clarify ambiguous or elusive gene expression patterns. Additionally, many of the modifications we present here can be applied directly to FISH protocols for other model organisms.

## Results and discussion

### Formamide bleaching increases signal intensity

Achieving maximal signal intensity in WISH requires balancing preservation of target mRNA with permeabilization of tissue to allow probe hybridization. Using the planarian WISH protocol established in [21] as a starting point, we began systematically testing modifications to improve signal sensitivity with the goal of improving detection of problematic transcripts by FISH. Because the TSA reaction used for fluorescent detection of transcripts rapidly proceeds to completion, we began by using alkaline phosphatase-based detection to directly compare the rate of development of various probes while varying conditions including fixation, bleaching, permeabilization, hybridization buffer, and hybridization temperature. We first tested the effects of these variations using readily detected transcripts, including the neoblast marker *smedwi-1*[24]; moderately detected transcripts, including a vacuolar ATPase B subunit we have identified as being upregulated in the intestine (*Smed-vatpaseB*), and the midline marker *Smed-slit-1*[25]; and with weakly detected transcripts including the hunchback-like transcription factor, *Smed-hb*, reported to be broadly expressed [15].

**Figure 1:**
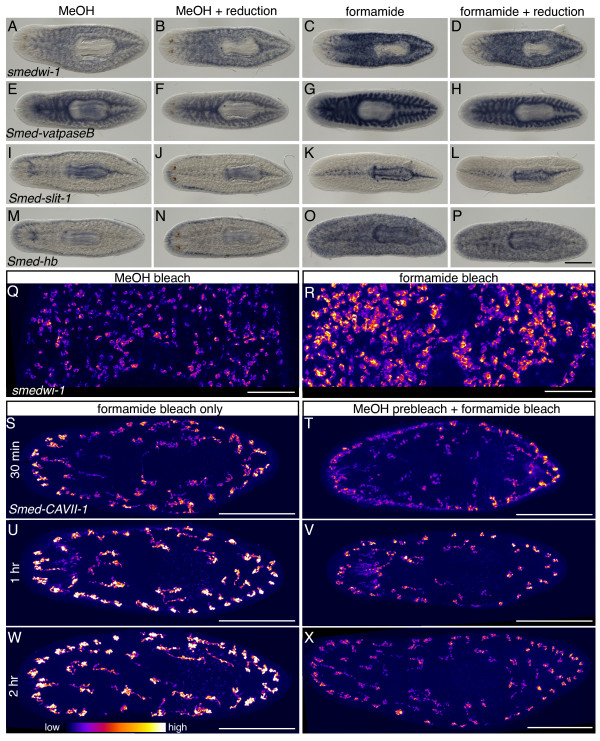
**Bleaching animals in formamide improves WISH and FISH signal.** (**A**-**P**) Chromogenic detection of *smedwi-1* (**A**-**D**), *Smed-vatpaseB* (**E**-**H**), *Smed-slit-1* (**I**-**L**), and *Smed-hb* (**M**-**P**) in planarians fixed with or without a reduction step and bleached either overnight in methanol or for 2 hours in formamide as indicated. Development for each probe was stopped at the same time to allow for direct comparison in signal intensity between conditions. (**Q**-**R**) Single confocal sections showing FISH for *smedwi-1* in the prepharyngeal region of planarians bleached overnight in methanol (**Q**) or for 2 hours in formamide (**R**). (**S**-**X**) Maximum intensity projections of whole planarians with FISH for *Smed-CAVII-1.* Planarians were either bleached in formamide alone for 30 minutes (**S**), 1 hour (**U**), or 2 hours (**W**), or pre-bleached overnight in methanol and then bleached again for 30 minutes (**T**), 1 hour (**V**), or 2 hours (**X**) in formamide. Confocal images were false colored in ImageJ using the fire look up table where blue = weak signal and white = strong signal. Scale bars: 500 μm (**A**-**P** and **S**-**X**); 100 μm (**Q**-**R**).

Most of the variations we tested had minimal impact on signal intensity. However, we found that replacing the overnight peroxide bleach in methanol with a short peroxide bleaching step in formamide dramatically reduced development time for all probes tested, indicating improved signal sensitivity (Figure 1A-P). For FISH, the increased signal intensity resulting from the short formamide bleach also improved the signal-to-noise ratio (Figure 1Q and R). Additionally, planarians bleached in formamide showed more consistent labeling of the prepharyngeal region, a densely packed area with typically reduced probe penetration, compared to methanol-bleached planarians, suggesting that tissue permeability was improved. We examined whether a reduction step [21], which was added to improve permeability of the prepharyngeal region, was required in formamide-bleached planarians. Surprisingly, we found that the reduction step slightly diminished signal intensity (Figure 1A-P). If peroxide bleaching in formamide functions to improve tissue permeability, signal intensity should gradually increase with longer bleaching times and eventually reach a maximum level of signal. Consistent with this, we noticed signal intensity improved dramatically after bleaching for 30 minutes, reaching a maximum between 1 to 2 hours incubation in formamide bleaching solution (Figure 1S, U, and W). Interestingly, the improved signal intensity resulting from bleaching in formamide is lost when animals are first bleached overnight in methanol (Figure 1T, V, and X). One possibility for this could be damage of target mRNAs during the long methanol bleaching step. However, when we compared unbleached animals to animals bleached overnight in methanol we observed similar signal intensity (not shown). Additionally, while planarians bleached overnight in formamide had slightly more diffuse signal, signal intensity was similar to animals bleached for two hours in formamide (not shown). These results suggest mRNA is relatively stable during an overnight peroxide bleach, and that pre-bleaching in methanol must mask the benefit of bleaching in formamide through some other mechanism. Interestingly, signal intensity for WISH increases slightly in zebrafish following peroxide bleaching in methanol [26]. Our finding of enhanced signal intensity with formamide-bleaching could be directly and broadly beneficial in improving WISH signal in other organisms, whether they are pigmented or not.

### Modified blocking and wash buffers dramatically improve signal specificity

One of the challenges in achieving high signal sensitivity for FISH is that the TSA reaction proceeds rapidly to completion and cannot be monitored and stopped when an optimal signal-to-noise ratio has been reached. Therefore, eliminating weak background staining is vital for optimal signal sensitivity when using the TSA reaction for FISH.

**Figure 2:**
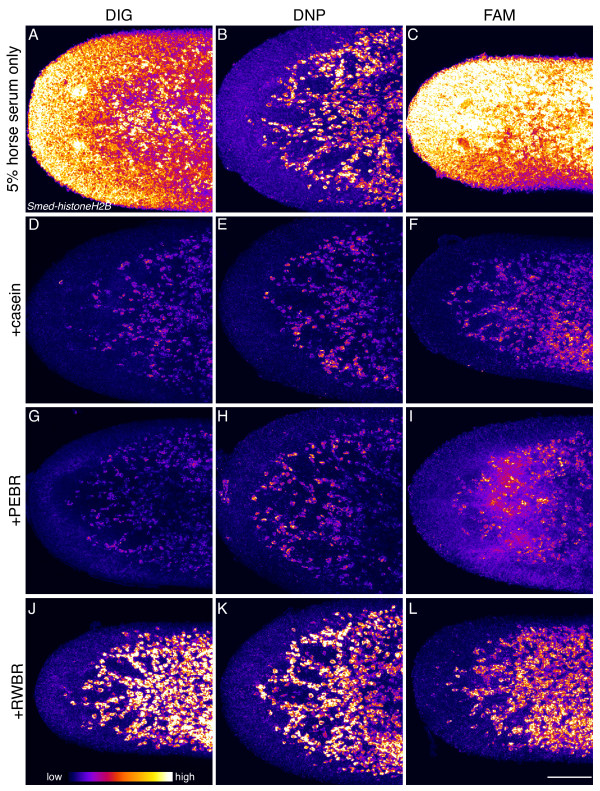
**Addition of blocking reagents greatly improves signal specificity for peroxidase-conjugated anti-hapten antibodies.** Planarians were hybridized with DIG-, DNP-, or FAM-labeled anti-sense RNA probes to *Smed-histoneH2B* (as indicated) and then blocked in buffer with either 5% horse serum alone (**A**, **B**, and **C**), or in 5% horse serum with 0.75% casein (**D**, **E**, and **F**), 0.75% PerkinElmer Blocking reagent (PEBR) (**G**, **H**, and **I**), or 0.75% Roche Western Blocking reagent (RWBR) (**J**, **K**, and **L**) prior to incubation with the appropriate peroxidase-conjugated anti-hapten antibody in the same blocking buffer. Images are maximum intensity projections of planarian heads imaged using identical settings and false colored in ImageJ using the fire look up table. Scale bar: 100 μm.

To further improve signal sensitivity we next examined different blocking and wash solutions. A variety of different blocking and wash solutions have been employed for FISH in other systems [21,26-28]. We began by comparing the effect of adding various reagents to the blocking buffer. Since different antibodies can respond differently to changes in blocking solution, we tested modified blocking solutions with anti-digoxigenin- (DIG), anti-dinitrophenol- (DNP), and anti-fluorescein- (FAM) antibodies conjugated to peroxidase. We found that addition of either casein or PerkinElmer Blocking Reagent (PEBR) improved the signal-to-noise ratio for most of the antibodies tested, but also led to slightly reduced signal intensity (Figure 2). Impressively, addition of Roche Western Blocking Reagent (RWBR) dramatically reduced background, particularly for the anti-DIG and anti-FAM antibodies, without significantly affecting signal intensity (Figure 2).

**Figure 3:**
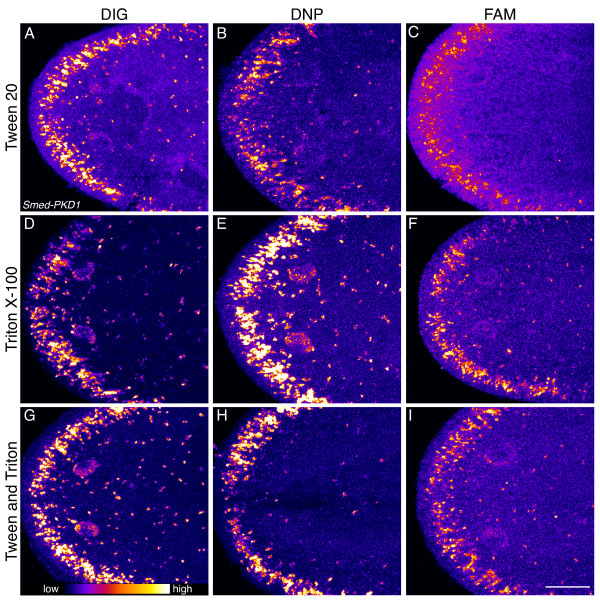
**Addition of Triton X-100 to blocking and wash buffers improves signal-to-noise ratio for peroxidase-conjugated anti-hapten antibodies.** Planarians were incubated, as indicated, with DIG-, DNP-, or FAM-labeled anti-sense RNA probes to *Smed-PKD1* and then blocked in 5% horse serum with 0.5% RWBR, incubated in anti-hapten antibody, and washed in Tris-sodium chloride buffer containing either 0.05% Tween (**A**, **B**, and **C**), 0.3% Triton X-100 (**D**, **E**, and **F**), or 0.05% Tween and 0.3% Triton (**G**, **H**, and **I**). Images are maximum intensity projections of planarian heads imaged using identical settings and false colored in ImageJ using the fire look up table. Scale bar: 100 μm.

Blocking and wash solutions for whole-mount FISH in a variety of animals differ in the use of maleic acid, phosphate, or Tris as the buffering component, but typically contain Tween 20 as a detergent [21,26-28]. We did not observe significant differences between the use of different buffering reagents, simplifying the protocol by reducing the number of stock solutions to prepare and allowing for the use of solutions that are more convenient to make or are already at hand. Significantly, we did find that altering the detergents present in the blocking and wash solutions further improved signal specificity. Addition of, or substitution with, 0.3% Triton X-100 resulted in a slight but noticeable improvement in signal (Figure 3). The benefit was especially pronounced with the anti-DIG and anti-FAM antibodies. There are relatively few peroxidase-conjugated anti-hapten (e.g. DIG, DNP, and FAM) antibodies available that are suitable for TSA, and the reagents described here are widely used for FISH in other model systems. While every model system presents its own unique requirements, the modified blocking solution we present here should have wide utility for the use of these anti-hapten antibodies in other organisms. Chromogenic WISH using alkaline phosphatase-based reagents has minimal background staining compared to FISH, and not surprisingly we observed little difference when RWBR or Triton X-100 was used for chromogenic detection (not shown).

### Quenching endogenous autofluorescence with copper sulfate

**Figure 4:**
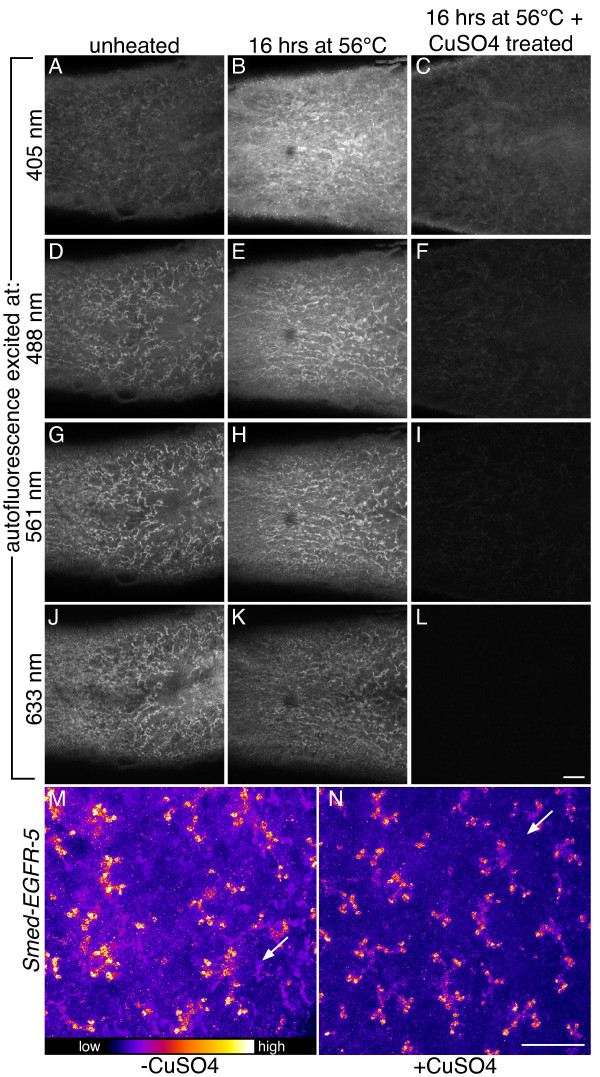
**Autofluorescence is reduced following treatment with copper sulfate.** (**A**-**L**) Single confocal sections of the prepharyngeal region of planarians excited with 405 nm (**A**, **B**, and **C**), 488 nm (**D**, **E**, and **F**), 561 nm (**G**, **H**, and **I**), and 633 nm (**J**, **K**, and **L**) laser lines. Planarians exhibit significant autofluorescence at several wavelengths (**A**, **D**, **G**, and **J**) that increases following a 16 hour incubation at 56°C (**B**, **E**, **H**, and **K**). Increased autofluorescence is significantly reduced at all wavelengths following a 1 hour incubation in copper sulfate solution (**C**, **F**, **I**, and **L**). (**M** and **N**) Maximum intensity projection of the dorsal posterior of a planarian following FISH for *Smed-EGFR-5* before (**M**) or after (**N**) copper sulfate quenching of autofluorescence. Arrows indicate the same position in M and in N. Scale bar: 100 μm.

Planarians exhibit autofluorescence over a broad range of wavelengths, and this feature has been used to distinguish newly regenerated tissues from more mature tissues [14]. When we compared the intensity of autofluorescence at various wavelengths between planarians incubated overnight in hybridization buffer at room temperature (Figure 4A, D, G, and J) or at 56°C (Figure 4B, E, H, and K) we noticed an increase in autofluorescence at several wavelength ranges in animals incubated at 56°C. As higher levels of autofluorescence reduce the signal-to-noise ratio, it can be difficult to distinguish real FISH signal from background autofluorescence, especially for low-abundance transcripts. One approach for improving the signal-to-noise ratio for FISH experiments is to use longer wavelength fluorophores for weakly detected transcripts, as autofluorescence tends to be stronger in the blue-to-green range of the spectrum.

An additional approach for improving signal sensitivity is to reduce or eliminate autofluorescence. While there are a variety of causes for autofluorescence, the broad range of autofluorescence in planarians, its increase following incubation at high temperatures, and its resistance to photobleaching (not shown) is similar to lipofuscin-based fluorescence observed in tissues of other animals [29,30]. Incubation in copper sulfate solution has been reported to quench lipofuscin-based autofluorescence [29,30]. To test the ability of copper sulfate to reduce background signal in planarians, we incubated heat-treated animals for 1 hour in copper sulfate solution (10 mM CuSO4, 50 mM ammonium acetate pH 5.0) and imaged using identical settings to the unheated and heat-treated samples. The copper sulfate treatment dramatically reduced autofluorescence at all wavelength ranges examined (Figure 4C, F, I, and L).

The nearly complete elimination of autofluorescence we observed was very encouraging. However, treatment with copper sulfate has been reported to quench some fluorophores [29]. To test whether the benefits of copper sulfate outweigh its potential harm to signal, we analyzed expression of *Smed-EGFR-5* (*EGFR-5*), which is detected at moderate levels in protonephridia [14], using TAMRA-conjugated tyramide before and after copper sulfate treatment. Prior to quenching, detection of *EGFR-5* in protonephridia was discernible, but autofluorescence in the secretory cells, which have a similar tubular pattern, complicated visualization of signal (Figure 4M). When we imaged the same animal after treatment with copper sulfate we had to increase the gain to achieve a similar level of brightness. However, the signal-to-noise ratio was dramatically improved, greatly facilitating visualization of *EGFR-5* (Figure 4N). The significantly enhanced signal-to-noise ratio we observed for *EGFR-5* highlights the utility of copper sulfate treatment for analyzing the expression pattern of transcripts with weak-to-moderate signals. Copper sulfate treatment should also be useful for multicolor fluorescence experiments, as we have noticed only minor quenching of DyLight 405-, FAM-, Cy3-, and DyLight 633-tyramides as well as Alexa488-conjugated secondary antibodies following copper sulfate treatment (not shown).

### Balancing signal sensitivity while preserving tissue morphology in regenerates

FISH analysis of planarians within the first few days following amputation presents a challenge, as the blastema tissue is particularly fragile and can be easily damaged by the aggressive treatments required to permeabilize mature tissues sufficiently. Therefore, it is important to achieve a balance during the permeabilization steps that allows for relatively even penetration of probe into mature tissues without excessively damaging blastema tissue. One strategy for accomplishing this is to adjust Proteinase K concentration and incubation time until a satisfactory result is obtained. Additionally, while experimenting with alternative methods for permeabilizing planarians, we noticed that heat-induced antigen retrieval (HIAR) resulted in slightly weaker signal in intact planarians, but allowed for consistent and even labeling throughout the animal while causing less damage to superficial layers compared to Proteinase K treatment (not shown).

**Figure 5:**
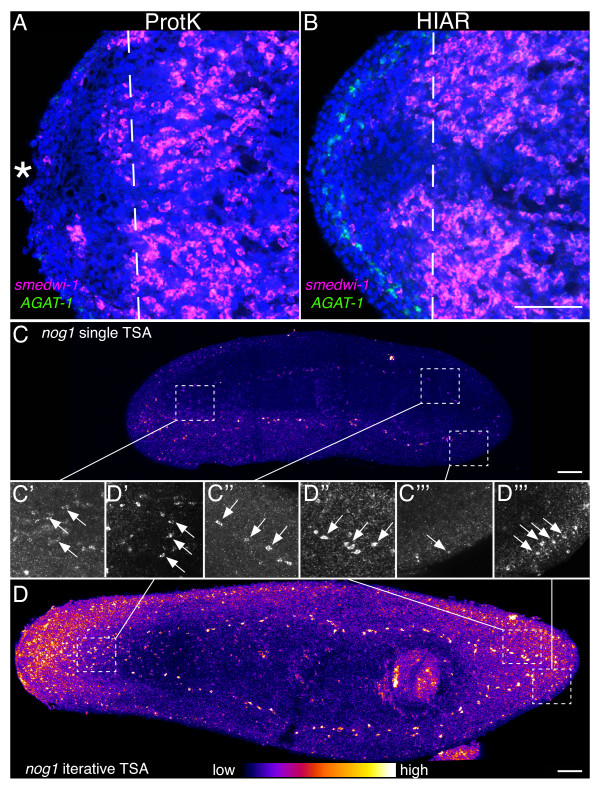
**Improved signal intensity in regenerates and for low-abundance transcripts*.*** (**A**-**B**) Single confocal sections of three day regenerating planarians labeled with DAPI (blue), the neoblast marker *smedwi-1* (magenta), and the differentiating cell marker *Smed-AGAT-1* (green). While gene expression is detectable in the intact tissue of planarians permeabilized with Proteinase K (**A**) or following HIAR (**B**), the superficial layers of the blastema (asterisk), including the *AGAT-1*-expressing cells, are lost following Proteinase K treatment. Dashed line denotes plane of amputation. (**C**-**D**) Maximum intensity projection of animals hybridized with DIG-labeled RNA probes for *Smed-nog1* with a standard single TSA using TAMRA-conjugated tyramide (**C**) or iterative TSA first using DNP-tyramide followed by washing, incubation with peroxidase-conjugated anti-DNP antibody, and a second TSA reaction with TAMRA-conjugated tyramide (**D**). (**C’**-**D”’**) Maximum intensity projections of regions from the planarians in C and D, including the brain (**C’** and **D’**), ventral nerve cord (**C”** and **D”**) and the lateral margin (**C”’** and **D”’**). Scale bars: 100 μm.

We decided to see whether HIAR would achieve the desired balance between permeabilization and preservation of tissue morphology in regenerates. For this purpose we performed FISH on planarians fixed three days after amputation using the neoblast marker *smedwi-1*[24] and a differentiation marker, *Smed-AGAT-1* (*AGAT-1*) [31], which is expressed in superficial cells just basal to the epidermis. Planarians were processed in parallel and were either permeabilized with Proteinase K treatment followed by post-fixation, or were boiled in sodium citrate buffer for 10 minutes and then incubated in Phosphate Buffered Saline [32] containing 0.3% Triton X-100 and 1% SDS for 20 minutes at room temperature. Single confocal sections of central focal planes reveal strong and even labeling of neoblasts with *smedwi-1* in intact tissues for both treatments (Figure 5A and B, magenta). However, blastema morphology was better preserved in planarians treated by HIAR, as the epidermis and superficial layer of *AGAT-1*-expressing cells are retained, and density of nuclei is higher. We also observed less damage to blastema tissue in planarians treated by HIAR in chromogenic WISH (not shown). In addition to the benefit of HIAR for FISH of regenerating planarians, this method may also be useful for immunostaining following FISH as Proteinase K treatment can destroy epitopes for some antibodies.

### Enhancing signal intensity through iterative TSA

For particularly low-abundance transcripts, gene expression patterns can be especially difficult to determine due to low signal intensity. While new and more sensitive imaging systems are vastly improving the ability to image weak signals, it can still be difficult to rapidly screen expression patterns in multiple samples by epifluorescence to identify animals or regions on which to focus imaging efforts. For example, *Smed-nog1* (*nog1*) is weakly to moderately detected in portions of the central nervous system, around the body margins, at the base of the pharynx, and at the mouth [23,33]. *nog-1* FISH signal following a conventional single TSA is challenging to discern when viewed by eye under epifluorescence, but yet is capable of being detected by confocal microscopy (Figure 5C). In an attempt to boost signal intensity for *nog1* we performed TSA first with DNP-conjugated tyramide, then incubated with peroxidase-conjugated anti-DNP antibody followed by a second, iterative, TSA with fluorophore-conjugated tyramide. In the first reaction signal is amplified by covalently depositing multiple DNP-tyramide molecules near the site of antibody binding. The signal is then further amplified by localizing additional peroxidase-conjugated antibody to the sites of DNP deposition and then performing an additional amplification with fluorophore-conjugated tyramide, which can then be visualized. When we performed iterative TSA for *nog1* we noticed a dramatic increase in signal intensity that greatly facilitated observation (Figure 5D). At higher magnification it is easy to identify *nog1*-positive cells near the cephalic ganglia in animals processed with iterative TSA (Figure 5D’), whereas with single TSA, signal is detected just above background (Figure 5C’). *nog1* detection is stronger in the ventral nerve cords, and easily observed following either single or iterative TSA (Figure 5C” and D”). The weak expression in cells around the body margin is almost undetectable following single TSA (Figure 5C”’), but after iterative TSA, *nog1*-expressing cells are easily identified (Figure 5D”’). The improved specificity from the optimized blocking and wash buffers has been particularly beneficial to iterative TSA, as minor background from non-specific antibody binding is greatly amplified with this method. While the extensive washing following TSA with DNP-conjugated tyramide appears critical, we have had success deploying this technique in multicolor FISH experiments without greatly extending the length of the experiment (see Additional file 1 for details).

### Azide effectively quenches peroxidase activity without inhibiting subsequent gene detection in multicolor FISH

**Figure 6:**
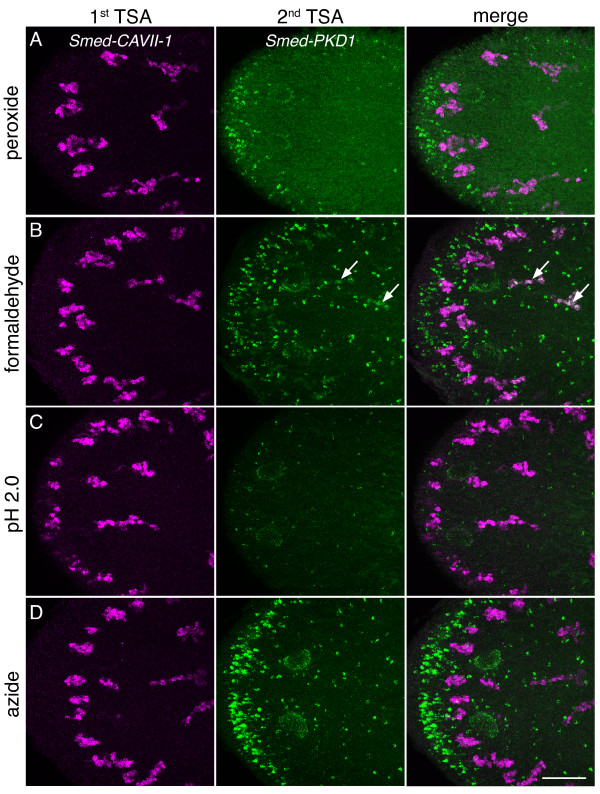
**Azide effectively quenches residual peroxidase activity in multicolor FISH.** (**A**-**D**) The efficacy of several peroxidase quenching compounds was compared by performing multicolor FISH for *Smed-CAVII-1* (expressed at high levels in protonephridia) and *Smed-PKD1* (expressed at moderate levels in a subset of neurons). *CAVII-1* expression was detected, then residual peroxidase activity was quenched by incubating samples for 45 min with either 2% hydrogen peroxide (**A**), 4% formaldehyde (**B**), glycine buffer pH 2.0 (**C**), or 100 mM azide (**D**). Finally, a second TSA reaction to detect expression of *PKD1* was performed. Arrows indicate signal from residual peroxidase activity of *CAVII-1* in the green channel. Scale bar: 100 μm.

**Figure 7:**
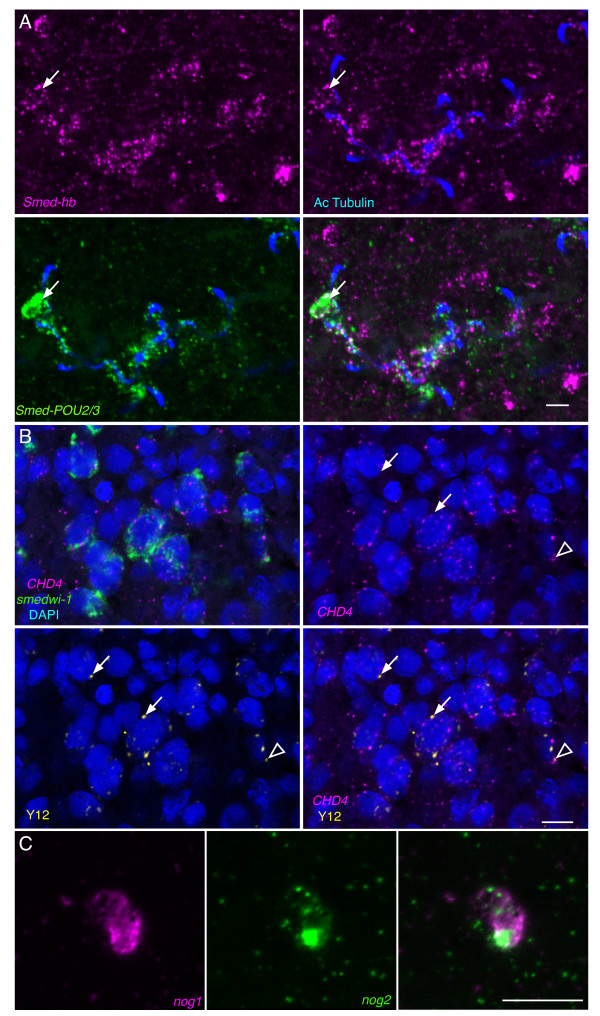
**Modified FISH protocol for determining expression patterns of problematic genes.** (**A**) Maximum intensity projection showing enriched expression of *Smed-hb* (magenta) in ciliated protonephridia labeled by anti-acetylated α-Tubulin (blue) and *Smed-POU2/3* (green). A subset of *POU2/3* positive putative protonephridial progenitor cells in close association with ciliated protonephridia coexpress *hb* (arrow). (**B**) Single confocal section showing coexpression of *Smed-CHD4* (magenta) and *smedwi-1* (green) in intact planarians stained with DAPI (blue) and the chromatoid body marker, Y12 (yellow). Most chromatoid bodies lack *CHD4* mRNA (closed arrows). However, there are occasional chromatoid bodies in close association with *CHD4* puncta (open arrowhead). (**C**) Maximum intensity projection of a cell expressing *Smed-nog1* (magenta) and *Smed-nog2* (green). Scale bar: 10 μm.

Besides providing excellent spatial resolution of gene expression, FISH has particular utility in determining the expression of genes relative to one another. For multicolor FISH to be effective, peroxidase activity of the first antibody used must be quenched effectively prior to subsequent detection rounds without leading to progressive degradation of sample. A number of methods have been described for inactivating peroxidase activity in multicolor FISH experiments, including incubation with hydrogen peroxide [21], fixation with formaldehyde [34], incubation in low pH buffer [35], and treatment with azide [36]. While incubation with hydrogen peroxide is more widely used, there does not seem to be a consensus on which method is the most effective. Therefore, we decided to compare directly several methods to determine which was the most effective at inactivating peroxidase activity and least detrimental to detection of subsequent gene expression patterns in planarians. For this we performed multicolor FISH for two non-overlapping genes; *Smed-CAVII-1* (*CAVII-1*), which is detected at high levels in the protonephridial system [37], and a homolog of Polycystin 1, *Smed-PKD1* (*PKD1*), which is detected at moderate levels in a subset of neurons in the anterior margin and in the sub-epidermal nervous plexus (Figure 3). We detected *CAVII-1* expression first, then incubated in either 2% hydrogen peroxide (Figure 6A), 4% formaldehyde (Figure 6B), 100 mM glycine pH 2.0 (Figure 6C), or 100 mM sodium azide (Figure 6D) for 45 minutes to quench peroxidase activity, before finally detecting *PKD1* expression. Peroxidase quenching using hydrogen peroxide has been widely used in planarians [21]. However, we find that while hydrogen peroxide does effectively quench peroxidase activity it also led to an increase in background fluorescence for *PKD1* (Figure 6A). More recently formaldehyde fixation has been used in planarian multicolor FISH experiments [34], but while signal sensitivity for *PKD1* was unaffected, there was clearly residual peroxidase activity from detection of *CAVII-1* (Figure 6B arrows). Inactivation of peroxidase activity using low pH has been described in other systems [35,36], and was effective at eliminating residual peroxidase activity from detection of *CAVII-1* (Figure 6C). However, it also greatly reduced signal intensity for *PKD1*. We found that incubation in 100 mM azide was effective at inactivating peroxidase activity without reducing signal sensitivity (Figure 6D), particularly when determining coexpression of low abundance transcripts (Figure 7). While the utility of azide in quenching peroxidase activity has been examined [36], it has not been widely used for multicolor FISH or immunohistochemistry using TSA. One possibility for this is that it has typically been used at lower concentrations where it may be less effective compared to other methods. Our results suggest that use of high concentrations of azide to inactivate peroxidase activity could prove useful for multicolor FISH experiments as well as for immunostaining experiments using TSA in other model organisms.

### Application of the *in situ* hybridization modifications to detect difficult transcripts

With the improved signal sensitivity we were able to achieve with our modifications to the planarian FISH protocol, we sought to resolve the expression patterns of a few genes with unclear expression. The transcription factor *Smed-hb* (*hb*) is required for normal maintenance and regeneration of protonephridia [15,38]. However, based on the published expression pattern it is unclear whether *hb* is expressed in protonephridia. Therefore, whether it acts autonomously in protonephridia or non-autonomously remains unresolved. We used *hb* as a representative problematic transcript for optimization of our FISH protocol, and found dramatically improved signal sensitivity particularly following formamide bleaching (Figure 1O). While we did observe broad expression of *hb* following chromogenic detection, we noticed staining of tubular structures consistent with protonephridial expression as well as stronger, punctate expression throughout the animal. To determine whether the tubular expression coincides with protonephridia, we performed FISH for *hb* and immunostained with anti-acetylated α-Tubulin antibody, which labels cilia in the lumen of protonephridial tubules [39-41]. We observed clear signal for *hb* surrounding ciliated protonephridia, consistent with *hb* function being required autonomously in protonephridial cells (Figure 7A). Scimone et al. (2011) have described a population of protonephridial progenitor cells defined by overlapping expression of protonephridial transcription factors [15]. To examine whether the punctate staining we observed for *hb* might represent protonephridial progenitor cells, we performed double FISH experiments with *hb* and several protonephridial transcription factors, including *Smed-POU2/3*, and found that a few of the *hb*-positive cells also expressed *POU2/3* (Figure 7A). While we did observe expression of *hb* outside of the protonephridia, these results support the possibility that *hb* function may be required in protonephridial progenitor cells and mature cell types.

The planarian gene *Smed-CHD4* (*CHD4*), is a homolog of the chromatin remodeling gene *CHD4/Mi-2*, and is required for the normal differentiation of neoblasts [22]. *CHD4* has been reported to be broadly expressed and enriched in the central nervous system [22]. Consistent with *CHD4* expression in neoblasts, mesenchymal expression is reduced following lethal irradiation, and *CHD4 in situ* hybridization of sorted cells results in labeling of a significant fraction of neoblast subtypes [22]. While these data provide compelling evidence that neoblasts express *CHD4*, we wanted to see if we could verify coexpression of *CHD4* with other neoblast markers in intact animals. For this we performed multicolor FISH for *CHD4* and *smedwi-1*. While we noticed broad expression of *CHD4* throughout the animal, there was clear punctate expression of *CHD4* in the cytoplasm of neoblasts (Figure 7B). The punctate, cytoplasmic localization of *CHD4* RNA in neoblasts is reminiscent of transcripts present in ribonucleoprotein complexes called chromatoid bodies that are believed to be important sites of post-transcriptional regulation [42]. To examine the possibility that *CHD4* mRNA localizes to chromatoid bodies we immunostained with the monoclonal antibody Y12 [43], which recognizes symmetrical dimethylarginine in proteins associated with chromatoid bodies [44]. While we occasionally observed *CHD4* puncta (magenta) near chromatoid bodies (yellow) (Figure 7B open arrowheads), we rarely observed overlap between Y12 immunostaining (Figure 7B arrows) and *CHD4* FISH signals. This observation suggests that the punctate signals observed represent subcellular localization of transcripts to cytoplasmic regions other than chromatoid bodies.

The expression patterns for several members of the *noggin* gene family in planarians have been described [23]. *Smed-nog2* (*nog2*) is one of a few *noggin* gene family members whose mRNA distribution remained elusive despite confirmation of expression by Reverse Transcription-quantitative PCR. While knockdown of either *nog1* or *nog2* alone yields no phenotype, *nog1*; *nog2* double knockdown leads to a dorsalization phenotype [45], further bolstering the likelihood that *nog2* is expressed. In single FISH experiments we were able to detect *nog2* expression (Additional file 2), and observed a pattern similar to that of *nog1*. We were curious to determine whether *nog1* and *nog2* are coexpressed, or whether different cells contribute either *nog1*or *nog2* to regulate dorsoventral polarity. To examine this we performed double FISH for *nog1* and *nog2*. Signal for *nog2* was clear but significantly weaker than for *nog1*. We found only a small percentage of *nog1-*positive cells that also expressed *nog2* in the body margin (Figure 7C). The more limited expression pattern for *nog2* compared to *nog1* could be real, or may indicate that expression of *nog2* in some cells is below the limit of detection. Despite the latter possibility, our ability to at least partially detect gene expression for a gene that has been refractory to analysis highlights the utility of the modifications we have established for this protocol.

## Conclusions

The FISH protocol we present here represents a significant improvement in signal sensitivity for the rapidly growing planarian field. Additionally, the modifications we have developed may be beneficial for FISH in other model systems. The short formamide bleaching step seems to improve tissue permeabilization properties, and therefore may be a useful addition to WISH protocols in other organisms where removal of pigment may or may not be a necessary step. Tissue autofluorescence is not unique to planarians, and while there are multiple causes of autofluorescence, treatment with copper sulfate may provide similar benefits for FISH in other organisms. The peroxidase-conjugated antibodies used here are commonly employed for FISH in numerous model systems, therefore the modified blocking and wash buffers described here will no doubt improve FISH sensitivity in other organisms, and the azide treatment will be directly applicable in other multicolor FISH experiments.

For large/complex organisms it can be difficult to rapidly identify cells or tissues expressing low-abundance transcripts. To improve initial screening we have developed methods for iterative TSA, which significantly improve signal intensity, facilitating identification of expression domains. Finally, while HIAR methods have been extensively used for tissue permeabilization in immunofluorescence protocols [46,47], we show that this method can also be applied for FISH, enabling sufficient permeabilization with improved morphology of fragile tissues. Additionally, this method may be widely useful in allowing for the use of some antibodies that are sensitive to Proteinase K treatment following FISH. Together, the enhanced signal specificity resulting from the modifications presented here will no doubt be useful for shedding light on how planarians achieve their remarkable regenerative capacity.

## Methods

### Planarian culture

Asexual *Schmidtea mediterranea* clonal line CIW4 [37] was maintained in the dark at 20°C in deionized water containing 0.5 g/L Instant Ocean Sea Salts. Animals were fed pureed organic calf liver 1–2 times per week and starved for 1 week before use.

### RNA probes

Hapten-labeled anti-sense RNA probes were generated from *in vitro* transcription reactions containing either DIG-12-UTP (Roche), DNP-11-UTP (PerkinElmer), or FAM-12-UTP (Roche) according to the manufacturer’s suggested protocol (Roche). DNA template for the *in vitro* transcription reaction was generated by PCR amplifying sequences from gene clones obtained from either the *S. mediterranea* EST Database [48] or from cDNA clones generated using standard methods. Primers and unincorporated nucleotides were removed from PCR products using a DNA clean and concentrator kit (Zymo Research) prior to use as template in transcription reactions. Probes were precipitated using LiCl/ethanol according to the manufacturer’s suggested protocol (Roche) and resuspended in 50 μl RNAse-free water. Probe quality and concentration were assessed on a 1% agarose gel and using a NanoDrop ND-1000 spectrophotometer. Probe concentration was adjusted to 50 ng/μl by adding hybridization buffer (see Additional file 1) and probes were stored at −20°C. In some cases, FAM-labeled probes were further purified using a sephadex G-50 quick spin column (Roche) according to the manufacturer’s recommendations.

### Animal pretreatment and hybridization

Unless otherwise noted, asexual planarians 1–5 mm in length were processed for WISH essentially as described [21] with the following significant modifications: the reduction step prior to dehydration was omitted. Bleaching was performed for 2 hours in formamide bleaching solution (1.2% H_2_O_2_, 5% formamide, and 0.5xSSC [32]). For regenerating planarians, the Proteinase K/post fixation steps were replaced with a 10 minute boiling step in 10 mM sodium citrate pH 6.0 with 0.05% Tween20, followed by a 20 minute room temperature incubation in PBSTx (Phosphate Buffered Saline [32], 0.3% Triton X-100) with 1% SDS. Blocking and antibody incubation for peroxidase-conjugated anti-digoxigenin (1:2,000 [Roche]), anti-fluorescein (1:2,000 [Roche]), and anti-dinitrophenol (1:300 [PerkinElmer]) were performed with 5% horse serum and 0.5% RWBR in TNTx (100 mM Tris pH 7.5, 150 mM NaCl, 0.3% Triton X-100). For chromogenic detection using alkaline phosphatase-conjugated anti-digoxigenin antibody (1:2,000 [Roche]), antibody incubation and blocking were performed with 5% horse serum in TNTx, and post-antibody washes were with TNTx prior to development as described in [21].

### Immunofluorescence

Ciliated protonephridia were labeled with anti-acetylated α-Tubulin antibody (6-11B-1, Santa Cruz Biotech) diluted 1:1,000 with 5% horse serum and 0.5% RWBR in TNTx. Chromatoid bodies were labeled with the monoclonal antibody, Y12 (NeoMarkers) diluted 1:250 with 0.6% IgG-free BSA and 0.45% fish gelatin in PBSTx as described in [42].

### TSA reaction

Tyramide conjugates were synthesized as described [49] from N-hydroxy-succinimidyl-esters of 5/6-carboxyfluorescein (Pierce), 5-(and-6)-carboxytetramethylrhodamine (Molecular Probes), DyLight 633 (Pierce), and 6-(2,4-dinitrophenyl) amino hexanoic acid (Molecular probes). Tyramide signal amplification was performed by incubating planarians for 10 min in fluorophore-conjugated tyramide diluted 1:250–1:500 in 100 mM borate buffer pH 8.5, 2 M NaCl, 0.003% H_2_O_2_, and 20 μg/ml 4-iodophenylboronic acid. For double FISH experiments, residual peroxidase activity was quenched by incubating for 45 minutes in 100 mM glycine pH 2.0 or in PBSTx containing either 2% H_2_O_2_, 4% formaldehyde, or 100 mM sodium azide.

### Microscopic visualization

Animals were cleared in 80% (v/v) glycerol and mounted on slides. Planarians developed using the chromogenic alkaline phosphatase substrate NBT/BCIP were imaged on a Leica M205A microscope equipped with a Leica DFC420 camera and a Leica TL 4000 RC base that was adjusted for Rottermann contrast. Fluorescent images were collected on a Carl Zeiss LSM710 confocal microscope running ZEN 2011. Images were processed using ImageJ 1.47f [50].

### Quenching autofluorescence

In cases where animal autofluorescence was significant, animals were gently removed from slides, washed in PBSTx, washed two times in deionized water, and incubated for 1 hour in freshly prepared 10 mM copper sulfate; 50 mM ammonium acetate pH 5.0. Following the copper sulfate quench, animals were washed two times in deionized water and then PBSTx before being cleared in 80% glycerol and remounted on slides.

## Abbreviations

WISH: Whole-mount *in situ* hybridization; FISH: Whole-mount fluorescent *in situ* hybridization; DIG: Digoxigenin; DNP: Dinitrophenol; FAM: Carboxyfluorescein; TAMRA: 5- and 6- carboxytetramethylrhodamine; HIAR: Heat-induced antigen retrieval; TSA: Tyramide signal amplification; PEBR: PerkinElmer Blocking Reagent; RWBR: Roche Western Blocking Reagent.

## Competing interests

The authors declare that they have no competing interests.

## Authors’ contributions

RSK conceived and conducted all of the experiments. Manuscript drafts were written by RSK. PAN participated in the study’s design and coordination and assisted with manuscript drafts. Both authors read and approved the final manuscript.

## Supplementary Material

Additional file 1 **Detailed FISH protocol for detection of low-abundance transcripts in planarians.** This file contains a step-by-step protocol for use in the laboratory. Click here for file

Additional file 2 ***Smed-nog2* FISH in planarian head.** This movie shows the distribution of *nog2*-expressing cells in the ventral region of the planarian head. Click here for file
