# Supplementary material for: In situ hybridization protocol for enhanced detection of gene expression in the planarian Schmidtea mediterranea
Source: BMC Dev Biol. 2013 Mar 12;13:8. doi: 10.1186/1471-213X-13-8 (PMC3610298; doi:10.1186/1471-213X-13-8)
Supplement: Additional file 1 — Detailed FISH protocol for detection of low-abundance transcripts in planarians. This file contains a step-by-step protocol for use in the laboratory. [file 1471-213X-13-8-S1.pdf]

Additional file 1: Detailed FISH protocol for detection of low abundance transcripts in planarians

Ryan S. King and Phillip A. Newmark

Step-by-step protocol..................................2
Stock solutions..5
TSA and formamide buffer cheat sheet.......8
Synthesis of tyramide conjugates...............9
Riboprobe synthesis..................................10
Reagents list..11
Notes...14
References...15

Step-by-step FISH protocol for planarians

*Unless otherwise mentioned, steps are performed at room temperature.
*Planarians are gently agitated throughout the protocol either on a nutator/rocker or by intermittent manual shaking unless noted otherwise.
*For wash/incubation steps remove >90% of previous solution before adding new or fresh solution.

Day 1: Animal fixation

1.1 Collect, wash, and transfer ~100 1-5 mm asexual planarians starved 1 week to a 15 mL conical tube. (For fewer animals volumes can be scaled down. For larger planarians, fewer animals can be processed or volumes can be scaled up)[1]

1.2 Remove excess Planarian salts, add 10 ml of 7.5% NAC solution, and gently rock for 5-10 min.

1.3 Remove NAC solution and fix for 15-20 min in 9 ml 4% Fixative with gentle agitation.

1.4 Wash in 10 ml PBSTx 2 times for 5 min.

1.5 Dehydrate in 10 ml 50% methanol for 5min, then in 10 ml 100% methanol 2 times for 5 min each and store animals at -20°C for 1 hr to a few months.

Day 2: Animal pretreatment and hybridization

2.1 Rehydrate samples by incubating in 10 ml 50% methanol for 5 min, then in PBSTx for 5 min.

2.2 Wash in 10 ml 1X SSC for 5 min.

2.3 Bleach animals by incubating in 15 ml freshly prepared Formamide-bleaching solution for 2 hrs under bright light[2].

2.4 Wash for 5 min in 10 ml 1X SSC.

2.5 Wash in 10 ml PBSTx 2 times for 5 min each.

2.6a For intact planarians incubate in 10 ml ProteinaseK solution for 10-15 min with gentle agitation (see below for permeabilization of regenerates using HIAR)[3].

2.7a Postfix the samples in 9 ml 4% Fixative for 10 min.

2.8 Wash in 10 ml PBSTx 2 times for 5 min each.

2.9 Transfer 3-5 animals each to 2.0 ml screw cap tubes or to small in situ baskets (Intavis) in either an Intavis InSitu Pro robot or a 48-well plate.

2.9 Incubate in 300 µl 1:1 Prehyb-PBSTx for 10 min.

2.10 Incubate in 300 µl Prehyb for 2 hrs at 56°C.

2.11 Hybridize in 300 µl Riboprobe mix for >16 hrs at 56°C.

Day 3: Posthyb washes and antibody incubation

3.1 Equilibrate samples by adding an equal volume of 2X SSCx and incubating at 56°C for 20 min. Alternatively, riboprobe mix can be removed and stored at -20°C for future use and planarians can be equilibrated by incubating in Wash hyb.

3.2 Wash at 56°C in 300 µl 2X SSCx 3 times for 20 min each.

3.3 Wash at 56°C in 300 µl 0.2X SSCx 4 times for 20 min each.

3.4 Return samples to room temp and wash in 300 µl TNTx 2 times for 10 min each.

3.5 Incubate animals in 300 µl Blocking solution for 1-2 hrs.

3.6 Remove blocking solution and incubate in 300 µl Antibody solution overnight at 4°C[4].

Day 4: Antibody washes and TSA reaction

4.1 Remove antibody solution and wash animals for 5 min, 10 min, and then 6 times for 20 min each in 300 µl TNTx.

4.2 Incubate in 300 µl freshly made Tyramide solution for 10min.

4.3 Wash in 300 µl TNTx 2 times for 5 min each.

4.4 For single FISH continue washing overnight in TNTx. For double/triple FISH, inactivate peroxidase activity by incubating in Azide solution for 45 min.

4.5 Wash peroxidase inactivated samples in TNTx 4 times for 10 min each.

4.6 Block in Blocking solution for 1 hr.

4.7 Incubate in Antibody solution overnight at 4°C.

4.8 For double/triple FISH repeat steps 4.1-4.7 as necessary. See below for iterative TSA.

Clearing, mounting, and quenching of autofluorescence

5.1 Following post tyramide washes, clear samples by incubating in 200 µl 80% glycerol solution for several hours.

5.2 Transfer animals to a slide, remove excess 80% glycerol solution, position, cover with a No. 1 weight coverslip containing clay feet at corners, apply gentle pressure at corners of coverslip until animals are slightly compressed, add fresh 80% glycerol to the slide at the edge of the coverslip until area between coverslip and slide is nearly full, and seal with clear nail polish.

5.3 Occasionally animal autofluorescence results in poor signal to noise. To reduce background autofluorescence gently remove animals from slides and wash with PBSTx for 10 min.

5.4 Remove PBSTx and wash in deionized water 2 times for 5 min.

5.5 To quench autofluorescence incubate in freshly prepared $CuSO_4$ solution for 1 hr.

5.6 Remove $CuSO_4$ solution and wash in deionized water 2 times for 5 min.

5.7 Wash planarians in PBSTx 2 times for 5 min, reclear in 80% glycerol solution, and remount on slides.

Permeabilization of regenerates by heat induced antigen retrieval (HIAR)

2.6b Replace PBSTx with NaCit solution and incubate in boiling water for 10 min[5].

2.7b Return samples to room temperature, replace NaCit solution with PBSTx + 1% SDS, and incubate at room temp for 20 minutes. (proceed to 2.8)[6]

Iterative TSA for enhanced signal intensity

-For a single gene, develop first with DNPx-tyramide, wash for 24-hours (no need to quench peroxidase activity), incubate with anti-DNP-HRP antibody, wash, and perform second amplification with a fluorophore-conjugated tyramide.

-For iterative TSA with multicolor FISH, wash time can be reduced by performing detection for the more strongly expressed genes between iterative TSA reactions. For example, detection of a low abundance transcript, *geneA*, with DNP labeled riboprobe and a higher abundance transcript, *geneB*, with DIG labeled riboprobe could be performed as follows: first, perform TSA for *geneA* with DNPx-tyramide followed by inactivation of peroxidase activity with azide; then, following a few short washes, incubate with anti-DIG-POD antibody and detect *geneB* expression by TSA using a fluorophore-conjugated tyramide; finally, following inactivation of peroxidase activity for *geneB* and a few short washes, incubate with anti-DNP-HRP antibody and perform the iterative TSA reaction with a second fluorophore-conjugated tyramide.

Stock Solutions

-unless noted otherwise, stock solutions are kept at room temperature

10X PBS: 1.37 M NaCl, 27 mM KCl, 100 mM Na_2HPO_4, and 20 mM KH_2PO_4. pH adjusted to 7.4, Diethyl pyrocarbonate (DEPC)-treated, and autoclaved.

1X PBS: 10X PBS stock diluted to 1X with deionized water

PBSTx: 1X PBS + 0.3% Triton-X 100

20X SSC: 3 M NaCl and 0.3 M Sodium Citrate. pH adjusted to 7.0, DEPC-treated, and autoclaved.

1X SSC: 20X SSC stock diluted to 1X with deionized water

2X SSCx: 20X SSC stock diluted to 2X + 0.1% Triton X-100

0.2X SSCx: 20X SSC stock diluted to 0.2X + 0.1% Triton X-100

Deionized formamide: Stir 1 liter formamide with 50 g of AG 501-X8(D) resin for 1hr, filter, aliquot, and store at -80°C.

Yeast RNA: yeast RNA is prepared by dissolving yeast RNA in DEPC-treated water for three days, extracting through phenol, phenol-chloroform, and chloroform. RNA is precipitated and resuspended in formamide to a concentration of 50 mg/ml, stored at -20°C.

Planarian salts: 0.5 g/L Instant Ocean Sea Salts in deionized water

50% methanol: equal volumes of 1X PBSTx and 100% Methanol

Prehyb: to 25 ml de-ionized formamide add 12.5 ml 20X SSC, 0.1 ml of 50 mg/ml Yeast RNA, 5 ml 10% (v/v) Tween-20, and bring up to 50 ml with DEPC-treated water. Store at -20°C[7].

Hyb: prehyb solution with 5% dextran sulfate (diluted from a 50% stock), stored at -20°C.

Wash hyb: equal volumes of 2X SSCx and wash hyb base (25 ml non-deionized formamide, 12.5 ml 20X SSC, 5 ml 10% (v/v) Tween-20, brought up to 50 ml with ddH_2O, and stored at -20°C).

TNTx: 0.1 M Tris pH 7.5, 0.15 M NaCl, and 0.3% Triton X-100 (for 1 L: 12.11g Tris Base, 8.77 g NaCl, 3 ml 100% Triton X-100. pH to 7.5 and filter sterilize).

4-IPBA: 20 mg/ml 4-iodophenylboronic acid in dimethylformamide (DMF), stored at -20°C[9].

TSA buffer: 2 M NaCl; 0.1 M Boric acid, pH 8.5; filter sterilized and stored at 4°C[10].

80% glycerol solution: 80% (v/v) glycerol; 10 mM Tris, pH 7.5; 1 mM EDTA

NaCit solution: 10 mM sodium citrate pH 6.0; 0.05% Tween 20, stored at 4°C.

<u>The following solutions should be made just prior to use</u>

7.5% NAC solution: 7.5% (w/v) N-acetyl-L-cysteine dissolved in PBS

4% Fixative: 1 ml 36.5% formaldehyde solution in 8 ml PBSTx

Formamide-bleaching solution: 5% non-deionized formamide, 0.5X SSC, and 1.2% H_2O_2. **Caution** at high concentrations formamide and H_2O_2 undergo a violent reaction. Always dilute these reagents into the water before mixing.

ProteinaseK solution: to 9.9 mL PBSTx add 0.1 mL 10% (w/v) SDS and 5 µl 20 mg/ml proteinaseK.

Riboprobe mix: denature riboprobes at 80°C for 5 min, chill on ice, and dilute 1:10 to 1:500 (final concentration of ~0.1-5 ng/µl) in Hyb solution. A final probe concentration of ~1ng/µl is a reasonable starting point[8].

Blocking solution: 5% horse serum and 0.5% Roche Western Blocking Reagent (RWBR stock solution is at 10%) diluted in TNTx.

Antibody solution: anti-DIG-POD (1:2,000), anti-DNP-HRP (1:300), or anti-Fluorescein-POD (1:2,000) diluted in blocking solution.

Tyramide solution: fluor-tyramide (1:250-1:500), 4-IPBA (1:1,000), and H_2O_2 (0.003%) in TSA buffer.

Azide solution: 100 mM sodium azide in PBSTx

$CuSO_4$ solution: 10 mM $CuSO_4$; 50 mM ammonium acetate, pH 5.0

Quick reference sheet for scaling quantities of formamide bleaching solution and tyramide solution

Formamide Bleaching Solution

Reagent	1 mL	5 mL	10 mL	15 mL	20 mL	30 mL	40 mL	50 mL
**H_2O**	0.885	4.425	8.85	13.275	17.7	26.55	35.4	44.25
**Formamide**	0.05	0.25	0.5	0.75	1	1.5	2	2.5
**20x SSC**	0.025	0.125	0.25	0.375	0.5	0.75	1	1.25
**30% H_2O_2**	0.04	0.2	0.4	0.6	0.8	1.2	1.6	2

Caution at high concentrations formamide and H_2O_2 undergo a violent reaction. Always dilute these reagents into the water before mixing.

Tyramide Signal Amplification Buffer Cheat Sheet

Incubate for 10 min with tyramide solution.

Reagent	1 ml	2 ml	3 ml	4 ml	5 ml	6 ml	7 ml	8 ml
TSA buffer[1]	1	2	3	4	5	6	7	8
0.5% H_2O_2[2]	0.006	0.012	0.018	0.024	0.030	0.036	0.042	0.048
4IPBA[3]	0.001	0.002	0.003	0.004	0.005	0.006	0.007	0.008

Reagent	9 ml	10 ml	11 ml	12 ml	13 ml	14 ml	15 ml	20 ml
TSA buffer[1]	9	10	11	12	13	14	15	20
0.5% H_2O_2[2]	0.054	0.060	0.066	0.072	0.078	0.084	0.090	0.12
4IPBA[3]	0.009	0.010	0.011	0.012	0.013	0.014	0.015	0.02

[1] Stock TSA buffer is 2 M NaCl, 100 mM Borate buffer pH 8.5 filter sterilized stored at 4°C.
[2] to 492 µl TSA buffer add 8.5 µl 30% H_2O_2
[3] Stock 4-Iodophenylboronic acid (4IPBA) is at 20 mg/ml in DMF.

FAM, TAMRA, and DNPx tyramides can be used at ~1:500 dilution
Dye Light 633 diluted 1:250 seems to give the best signal to noise for most applications.

Synthesis of Tyramide conjugates

The following protocol is adapted from [1]. Note: N-hydroxy-succinimydyl-esters are light sensitive and prone to hydrolysis, therefore it is best to use fresh, high quality anhydrous N,N-Dimethylformamide (DMF) and protect from light.

1) Prepare tyramine stock by dissolving tyramine hydrochloride to 10 mg/ml in DMF containing 10 µl/ml triethylamine.
2) Prepare fluor-conjugated NHS ester by dissolving to 10 mg/ml in DMF.
3) Add tyramine stock to fluor-conjugated NHS ester (see below).
4) Incubate at room temperature protected from light for 2 hours.
5) Dilute fluor-conjugated tyramide with 100% ethanol to 1 mg/ml (see below).
6) Aliquot and store at -20°C. (FAM- and TAMRA-tyramides are stable for at least a few years, DyLight 633 is stable for at least six months).

Dye-NHS	Dye-stock	µl 10mg/mL tyramine	ml EtOH for 1mg/mL stock
FAM	100 mg	3,425 µl	86 ml
TAMRA	25 mg	750 µl	21.75 ml
DyLight 633	1 mg	15 µl	0.885 ml
DNPx	25 mg	1,000 µl	22.5 ml

FAM: 5/6 carboxyfluorescein succinimidyl ester
TAMRA: 5-(and-6)-carboxytetramethylrhodamine succinimidyl ester
DNPx: 6-(2,4-dinitrophenyl) amino hexanoic acid succinimidyl ester

Riboprobe synthesis

The following protocol is adapted from the manufacturer's suggestions (Roche). DNA template for the *in vitro* transcription reaction was generated by PCR amplifying target sequence from cDNA that includes either T3 or SP6 promoter sequences (T7 could also be used). Unincorporated primers and nucleotides were removed from the PCR product using a DNA clean and concentrator kit (Zymo research).

In Vitro Transcription reaction

2 µL 10x TXN buffer*
2 µL 10x RNA labeling mix**
1 µL rRNasin RNase inhibitor
2 µL RNA polymerase (T3, T7, or SP6 as appropriate)
up to 400 ng of clean and concentrated PCR product
bring up to 20 µl with RNAse free water

incubate at 37°C for 2hrs

*10x Transcription buffer or transcription buffer supplied with RNA polymerase
**10x RNA labeling mix: 10 mM C, A, and GTP; 6.5 mM UTP; and 3.5 mM DIG-11-UTP (Roche), DNP-11-UTP (PerkinElmer), or Fluorescein-12-UTP (Roche).

DNAse Treatment

Add 5 µl DNAse mix and incubate at 37°C for 15min

DNAse mix: 0.5 µL DNAseI, 0.5 µL 10x TxN Buffer, and 4 µL DEPC H_2O

Probe precipitation

Add 2.5 µL 4M LiCl and 75 µL 100% EtOH pre-chilled to -20°C[11]
Incubate at -60°C or below for 30 min to overnight
Centrifuge at >13,000 rcf for 15min, 4°C
Decant liquid
Wash pellet with -20°C 70% EtOH
Centrifuge at >13,000 rcf for 5min, 4°C
Decant liquid and air dry pellet for ~2-5 min
Resuspend in 50 µL DEPC treated water
Analyze on a 1% agarose gel, and quantify using a spectrophotometer[12]
Dilute probe to 50 ng/µl with hyb solution and store at -20°C.

Reagent	Manufacturer	Catalog #
4-Iodophenylboronic acid	Sigma	471933
48-well plates	VWR	62406-195
5-(and-6)-carboxytetramethylrhodamine succinimidyl ester	Molecular Probes	C-1171
5/6 carboxyfluorescein succinimidyl ester	Thermo Scientific/Pierce	46410
6-(2,4-dinitrophenyl) amino hexanoic acid succinimidyl ester	Molecular Probes	D2248
AG 501-X8(D) Resin Beads 20 – 50 mesh	BioRad	142-6425
Ammonium acetate	Sigma	A7330-500G
Anti-DIG-AP	Roche	11093274910
Anti-DIG-POD	Roche	11207733910
Anti-DNP-HRP	PerkinElmer	FP1128
Anti-Fluorescein-POD	Roche	11426346910
BCIP	Roche	1383221
Boric acid	Fisher	A73500
Chloroform	Fisher	C298-500
Copper(II) sulfate	Sigma	C1297-100G
Dextran sulfate	Sigma	D8906
Diethyl pyrocarbonate	Sigma	D5758
Digoxigenin-11-UTP	Roche	11209256910
DMF (N,N-Dimethylformamide)	Sigma	D4551
DNA clean and concentrator kit	Zymo Research	D4003
DNP-11-UTP	PerkinElmer	NEL555001
DyLight 633 NHS ester	Thermo Scientific/Pierce	46414
EDTA	Sigma	E5134-500G
Ethanol, 200 proof	Decon Labs	2716
Fluorescein-12-UTP	Roche	11427857910
Formaldehyde solution (36.5%)	EMD	FX0410-5
Formamide	Roche	11814320001

Reagent	Manufacturer	Catalog #
Glycerol	Fisher	G33-500
Hydrogen peroxide, 30%	Sigma	H1009
Instant Ocean Sea Salts	Dr. Foster & Smith	CD70441
Lithium chloride	Fisher	L121-500
Magnesium chloride tetrahydrate	Fisher	M87-100
Methanol	Fisher Chemical	A4124
N-acetyl-L-cysteine	Sigma	A7250
Nail polish (Wild Shine Clear)	Wet N' Wild/Walgreens	401A
NBT	Roche	1383213
Phenol	Roche	3117952001
Phenol-Chloroform	Roche	3117987001
Polyvinyl alcohol (PVA)	Sigma	P8136-250G
Potassium chloride	Fisher	P217-500
Potassium phosphate monobasic	Fisher	P285-500
Proteinase K (20 mg/ml)	Invitrogen	25530049
rATP, rCTP, rGTP, and rUTP	Promega	E6000
Roche Western Blocking Reagent	Roche	11921673001
RQ1 DNase, RNase-free	Promega	M6101
rRNasin RNase inhibitor	Promega	N2511
Sephadex G50 Quick Spin Columns	Roche	11274015001
Small in situ baskets	Intavis	12.340
Sodium azide	Fisher	S227-100
Sodium chloride	BP	S271-10
Sodium citrate dihydrate	Fisher	S279-500
Sodium phosphate dibasic anhydrous	Fisher	BP332-500
Triethylamine	Sigma	T0886
Tris Base	BP	152-1
Triton X-100	Fisher/BP	BP151-500

Reagent	Manufacturer	Catalog #
Tween 20	Fisher/BP	BP337-500
Tyramine hydrochloride	Sigma	T2879
Yeast RNA	Roche	10109223001

Notes

1. This protocol has largely been optimized for small intact asexuals. Incubation volumes for larger samples may need to be adjusted. Slightly longer ProteinaseK treatment may be needed to sufficiently permeabilize larger animals. For regenerates, see the HIAR modification.
2. Animals may appear slightly yellow or tan after bleaching. Often it is not necessary to bleach for any longer than two hours, and remaining pigment will be reduced during hybridization. For FISH we have not noticed any problems with imaging slightly pigmented samples. For colorimetric development with NBT/BCIP, we have had success with bleach times up to four hours to reduce pigmentation. Alternatively, we have also found that bleaching in formamide following colorimetric development does not reduce signal, and can further eliminate pigmentation.
3. ProteinaseK treatment is one of the most critical variables to optimize for in situ hybridization. We have noticed vastly different activities from various sources of ProteinaseK, and have noticed significant changes in aliquots of ProteinaseK following storage at -20°C over several months. The recommended 10-15 minute, 10 μg/ml ProteinaseK treatment is a good starting point, but ProteinaseK concentrations and incubation times may need to be adjusted depending on enzyme activity.
4. For colorimetric development using alkaline phosphatase-conjugated antibody, omit the Roche Western Blocking reagent from the Blocking and Antibody solutions. Incubate in anti-DIG-AP antibody (1:2,000 [Roche]). Perform washes in TNTx, and develop with NBT/BCIP according to [2].
5. Planarians often become translucent during heating.
6. Do not post-fix samples following HIAR. Proceed directly to wash and prehyb incubation steps.
7. Prehyb, hyb, and wash hyb solutions in other protocols contain 1 mg/ml yeast RNA. We have not noticed any issues for several probes tested when yeast RNA is reduced to 100 μg/ml or substituted with 100 μg/ml Salmon Sperm DNA.
8. Riboprobe concentration is a critical variable to optimize. We have noticed interesting changes in gene expression patterns when probe concentration is varied. Typically, lower probe concentration results in cleaner signal for both low abundance transcripts and robustly expressed genes. Additionally, we have had better success with longer riboprobes. We have not noticed issues with probe penetration for probes under 1.5 kbp, as direct comparison between full-length probe, hydrolyzed probe, or smaller subcloned fragments for a gene yielded similar staining patterns.
9. 4IPBA is essential for Alexa 568 and Dye Light 633 tyramides to work. While it does not seem to be required for Alexa 488, FAM, or TAMRA tyramides, it also does not inhibit the reaction at the above concentrations, and at least for TAMRA yields slight enhancement of signal
10. Addition of Tween 20 (0.1% final) and dextran sulfate (2% final) to the TSA base buffer yields a noticeable increase in signal for several tyramides tested, but also increased background, resulting in poorer signal-to-noise ratio, similar to use of commercial TSA

systems, such as the TSA plus system from PerkinElmer. Despite the slightly reduced signal, we prefer the signal-to-noise ratio achieved with the TSA buffer described.

11. For FAM-labeled riboprobes, LiCl/ethanol is very effective at precipitating small products and unincorporated FAM-UTP. We have used sephadex G50 quick spin columns (Roche) to further purify full-length probe from unincorporated nucleotides and shorter probe fragments, prior to probe precipitation. For the FAM-labeled probes we have examined, signal has been strong with minimal background whether probes were further purified with quick spin columns or not. However, for some probes yielding significant background, further probe purification may be beneficial.
12. We have often noticed that DNP-riboprobes yield very weak bands on a gel, despite similar concentrations as DIG or FAM riboprobes when quantified on a spectrophotometer. We have had success using DNP probes even when a band is not visible on a gel.

References

1. Hopman AHN, Ramaekers FCS, Speel EJM: **Rapid Synthesis of Biotin-, Digoxigenin-, Trinitrophenyl-, and Fluorochrome-labeled Tyramides and Their Application for In Situ Hybridization Using CARD Amplification**. *Journal of Histochemistry & Cytochemistry* 1998, **46**:771–777.

2. Pearson BJ, Eisenhoffer GT, Gurley KA, Rink JC, Miller DE, Sánchez Alvarado A: **Formaldehyde-based whole-mount in situ hybridization method for planarians.** *Dev Dyn* 2009, **238**:443–450.
